# Circulating MicroRNAs and Aerobic Fitness – The HUNT-Study

**DOI:** 10.1371/journal.pone.0057496

**Published:** 2013-02-28

**Authors:** Anja Bye, Helge Røsjø, Stian T. Aspenes, Gianluigi Condorelli, Torbjørn Omland, Ulrik Wisløff

**Affiliations:** 1 K.G. Jebsen Center of Exercise in Medicine, Department of Circulation and Medical Imaging, Faculty of Medicine, Norwegian University of Science and Technology, Trondheim, Norway; 2 Department of Cardiology, Division of Medicine, Akershus University Hospital, Lørenskog; and K.G. Jebsen Cardiac Research Centre, University of Oslo, Oslo, Norway; 3 Institute of Genetics and Biomedical Research, National Research Council of Italy, Milan, Italy; 4 Humanitas Research and Clinical Centre, Milan Italy; University of Toronto, Canada

## Abstract

Aerobic fitness, measured as maximal oxygen uptake (VO_2max_), is a good indicator of cardiovascular health, and a strong predictor of cardiovascular mortality. Biomarkers associated with low VO_2max_ may therefore represent potential early markers of future cardiovascular disease (CVD). The aim of this study was to assess whether circulating microRNAs (miRs) are associated with VO_2max_-level in healthy individuals. In a screening study, 720 miRs were measured in serum samples from healthy individuals (40–45 yrs) with high (n = 12) or low (n = 12) VO_2max_ matched for gender, age and physical activity. Candiate miRs were validated in a second cohort of subjects with high (n = 38) or low (n = 38) VO_2max_. miR-210 and miR-222 were found to be higher in the low VO_2max_-group (p<0.05). In addition, miR-21 was increased in male participants with low VO_2max_ (p<0.05). There were no correlations between traditional risk factors for CVD (blood pressure, cholesterol, smoking habit, or obesity) and miR-21, miR-210 and miR-222. DIANA-mirPath identified 611 potential gene-targets of miR-21, miR-210 and miR-222, and pathway analysis indicated alterations in several important signaling systems in subjects with low VO_2max_. Potential bias involve that blood was collected from non-fasting individuals, and that 8 performed exercise within 24 h before sampling. In conclusion, we found that miR-210, miR-21, and miR-222 were increased in healthy subjects with low VO_2max_. The lack of association between these three miRs, and other fitness related variables as well as traditional CVD risk factors, suggests that these miRs may have a potential as new independent biomarkers of fitness level and future CVD.

## Introduction

Aerobic fitness measured as maximal oxygen uptake (VO_2max_) is a good indicator of cardiovascular health, and a strong predictor of cardiovascular mortality in healthy individuals and in patients with cardiovascular disease (CVD) [1]–[4]. CVD is currently the predominant cause of morbidity and mortality in developed countries. To manage this pandemic, new and effective prevention strategies as well as new biomarkers of CVD risk are needed [5]. Based on the strong association between low aerobic fitness and CVD mortality, biomarkers associated with aerobic fitness may thus represent potential early markers of CVD.

Recently, microRNAs (miRs) have emerged as promising biomarkers of disease, as large amounts of stable miRs can enter the circulation [6]. miRs are short, endogenous, single-stranded, noncoding RNAs that negatively regulate gene expression [7]. More than 1000 miRs have so far been discovered, and their dysregulation have been associated with different pathologies like cancer and CVD [8]–[10]. Previously, increased circulating levels of miR-1 have been associated with myocardial infarction [11], circulating levels of miR-423 have been associated with heart failure [12], and circulating levels of miR-208 have been associated with myocardial damage in CVD [13]–[15]. Recently, several papers also report that circulating miRs may serve important endocrine functions in health and disease [16], [17].

To our knowledge, there are no previous studies reporting differences in circulating miRs in proportion to aerobic fitness level. As low aerobic fitness is an important risk factor of CVD, miRs that are upregulated in subjects with low aerobic fitness may represent early biomarkers of CVD. The aim of this study is to use new methods of miR-profiling to identify novel biomarkers associated with high and low VO_2max_.

## Methods

### Ethics Statement

The study is in conformity with Norwegian laws and the Helsinki declaration, and all participants signed a document of informed consent. The study was approved by the Regional Committees for Medical and Health Research Ethics (REC Central).

### Subjects

The third wave of the Nord-Trøndelag Health Study (HUNT3 Study) in Norway was carried out between 2006 and 2008. Participants in the present study attended a sub-study in HUNT3 designed to measure maximal oxygen uptake (VO_2max_) in healthy adult subjects and was called the HUNT Fitness Study [18]. Participants in the Fitness Study reported to be free from heart- or lung disease (details previously described [18]). Other exclusion criteria were cancer, pregnancy, or any other medical contraindication or orthopaedic limitation not permitting a maximal exercise stress test.

From the eligible 4631 participants who had completed a successful VO_2max_-test in the HUNT Fitness Study, 12 participants with high VO_2max_ and 12 participants with low VO_2max_ were selected for the initial screening study. All subjects were between 40 and 45 years old and were matched on gender, age (within 0.9 year between case and control), and equal levels of physical activity. In addition, the subjects were matched on the basis of having as similar as possible body mass index (BMI), blood pressure (BP), and serum levels of glucose, cholesterol and triglycerides. Thus, the only cardiovascular risk factor that was different between the cases and the controls was VO_2max_.

To verify the findings in a validation cohort, 76 new participants from the HUNT Fitness Study were selected (38 with low VO_2max_ and 38 with high VO_2max_) based on the same matching criterias as for the screening cohort.

### Anthropometric Measurements

Weight and height were measured on a combined scale DS-102 (Arctic Heating AS, Nttery, Norway), and BMI was calculated as weight divided by height squared (kg/m^2^). An individualized protocol was applied to measure VO_2max_ [19]. Oxygen uptake kinetics were measured directly by a portable mixing chamber gas-analyzer (Cortex MetaMax II, Cortex, Germany) with the participants wearing a tight face mask (Hans Rudolph, Germany) connected to the MetaMax II. The system has formerly been found to be valid [20]. Heart rate was measured by radio telemetry (Polar S610i, Polar Electro Oy, Finland). From the warm-up pace, the load was regularly increased when oxygen uptake kinetics flattened. Along with a respiratory quotient of 1.05 or higher, a maximal test was considered achieved when the oxygen uptake did not increase more than 2 ml/kg/min at the highest effort or before the participant disembarked the treadmill [21]. VO_2max_ was measured as litres of oxygen per minute (l/min) and subsequently calculated as VO_2max_ relative to body mass (ml/kg/min) and VO_2max_ scaled (ml/kg^0.75^/min).

### Blood Sampling and Standard Biochemical Analyses

Blood sampling was performed before the start of the exercise test. Standard biochemical analyses were performed on fresh venous non-fasting blood samples at Levanger Hospital, Norway. Non-fasting glucose was analysed by Hexokinase/G-G-PDH methodology reagent kit 3L82-20/3L82-40 Glucose, high-density lipoprotein (HDL) cholesterol by the Accelerator selective detergent methodology reagent kit 3K33-20 Ultra HDL, triglycerides by Glycerol Phosphate Oxidase methodology reagent kit 7D74 Triglyceride, alanine aminotransferase (ALAT) by NADH (with P-5′-P) methodology reagent kit 8D36-30 Alanine aminotransferase activated, aspartate aminotransferase (ASAT) by NADH (with P-5′-P) methodology reagent kit 8D37-30 Aspartate aminotransferase activated, and C-reactive protein (CRP) was analysed by the Areoset CRP Vario kit (all analyses from Abbott Diagnostics, Illinois, US). ALAT measurements below the detection limit (LoD) were assigned a concentration of 9 u/l, ASAT measurements below LoD a concentration of 7 u/l, and CRP levels below LoD were recorded as 0 mg/l.

### Questionnaire-based Information

Physical activity was registered based on the responses to a self-administered questionnaire [22]. The questionnaires included three questions: Question 1: “How frequently do you exercise?”, with the response options “Never” (0), “Less than once a week” (0.5), “Once a week” (1), “2–3 times per week” (2.5) and “Almost every day” (5). Question 2: “If you exercise as frequently as once or more times a week: How hard do you push yourself?” with the response options: “I take it easy without breaking a sweat or losing my breath” (1), “I push myself so hard that I lose my breath and break into sweat” (2) and “I push myself to near exhaustion” (3). Question 3: “How long does each session last?”, with the response options: “Less than 15 minutes” (0.1), “16–30 minutes” (0.38), “30 minutes to 1 hour” (0.75) and “More than 1 hour” (1.0). Each participant’s response to the above three questions (i.e. numbers in parentheses) were multiplied to calculate a physical activity index score (Kurtze score). As the second and third question only addressed people who exercised at least once a week, both “Never” and “Less than once a week” yielded an index score of zero. Participants with a zero score were categorized as inactive.

The Finnish Type 2 Diabetes Risk Score (FINDRISC) was calculated for all the participants based on lifestyle variables, family history and BMI. FINDRISC predicts the risk of being diagnosed with type 2 diabetes in the next ten years and requires no laboratory measurements [23]. We also calculated the Framingham Risk Score, which estimates the 10-year risk of having a myocardial infarction, and the NORRISK Score, which is optimized for the Norwegian population and estimates the 10-year risk of cardiovascular mortality [24], [25].

### miR Isolation

miRs were extracted from serum using the miRNeasy® Mini Kit (Qiagen, US). To control isolation efficiency, two syntetic spike-ins were added to the samples (UniSp2 and UniSp4). Briefly, 750 µl of a Qiazol mixture containing 1.25 µg/ml of MS2 bacteriophage RNA (RNA carrier not containing miRs) was added to 200 µl serum. The MS2 bacteriophage was added to increase the extraction of miRs. miRs were then extractet using chloroform, ethanol and spin columns. miRs were eluted in 50 µl of RNase-free water and stored in a −80°C freezer prior to analysis.

### Real-time Quantitative Polymerase Chain Reaction (RT-qPCR)

Fifteen µl RNA was reversely transcribed in 75µl reactions using the miRCURY LNA™ Universal RT microRNA PCR, Polyadenylation and cDNA synthesis kit (Exiqon, Denmark). cDNA was diluted 50× and assayed in 10 µl PCR reactions according to the protocol for miRCURY LNA™ Universal RT microRNA PCR. For the screening approach, all miRs were assayed once by RT-qPCR on the microRNA Ready-to-Use PCR, Human panel I and panel II. For the validation approach, selected candidate miR were analysed by the custom-made “Pick-and-mix” RT-qPCR system (Exiqon, Denmark). Negative controls excluding template from the reverse transcription reaction was included and profiled like the samples. The amplification was performed in a LightCycler® 480 RT-qPCR System (Roche, Switzerland) in 384 well plates. The amplification curves were analysed using the Roche LC software, both for determination of Cp (by the 2nd derivative method) and for melting curve analysis.

### miR Data Analysis

Screening of 720 miRs was performed in serum samples and the amplification efficiency was calculated using algorithms similar to the LinReg software. All assays were inspected for distinct melting curves and the Tm was checked to be within known specifications of the assay. Individual miRs had to be detected with Cp<37 and 5 Cp’s less than the negative control (blank) to be included in the data analysis. Data that did not pass these criteria were omitted from further analysis. Using SLqPCR on the screening cohort, the best normalizer was found to be the average of assays detected in all samples (global mean) and therefore data were normalized to the global mean (average – assay Cp). For the validation cohort, we normalized data to the level of miR-425, as miR-425 levels were expressed close to the global mean in the screening cohort and miR-425 previously has been found valid as a housekeeping miR [26].

### miR Target Prediction

The web-based computational tool DIANA-mirPath was used to identify molecular pathways potentially altered by the coordinated change in expression of the three fitness-miRs [27]. DIANA mirPath combines the prediction tool Targetscan 5.1 and the pathway tool KEGG (Kyoto Encyclopedia of Genes and Genomes). DIANA-mirPath was set to use TargetScan Human 5.1 to identify possible mRNA targets. The level of significance was set at p*<*0.05.

### Statistical Analysis

PASW Statistics 17.0 (IBM, US) was used for traditional statistical analyses. All statistical tests were two-sided, and p-values below 0.05 were considered statistically significant. Kolmogorov-Smirnov test was used to test for normality. One-Way ANOVA was used to compare variables between the high and the low VO_2max_ groups. Pearson’s correlation was used to study associations between normally distributed variables. Linear regression analysis was used to predict the contribution of miRs to the level of VO_2max_. Logistic regression was uset to determine the odds ratios for different predictors and outcomes.

## Results

**Figure 1:**
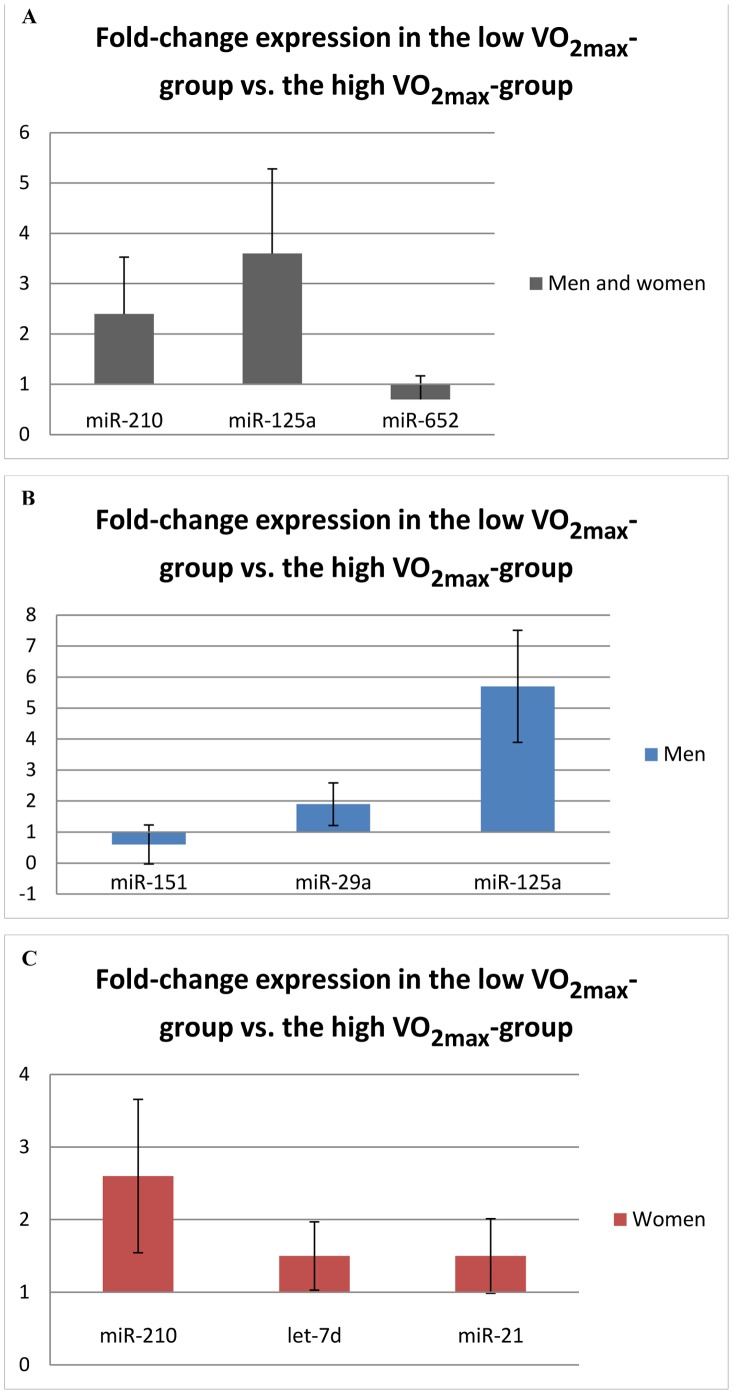
MicroRNAs with significantly different serum levels in individuals with high and low aerobic fitness in the screening cohort (p<0.05). A: In both men and women (n = 24), B: Men (n = 12), C: Women (n = 12). VO_2max_: Maximal oxygen uptake. Error bars represent 1 standard deviation.

The baseline characteristics of the populations are displayed in the Table 1. Out of the 720 analyzed miRs, 50 miRs were successfully assessed with sufficient signal in >80% of the samples and qualified for further analyses. Participants with low VO_2max_ had significantly higher levels of miR-210 and miR-125a compared to participants with high VO_2max_ in the screening cohort (ddCp>+/−0.50, p<0.01) (Figure 1A). In addition, subjects with low VO_2max_ had significantly lower serum levels of miR-652 (p<0.001) (Figure 1A). In male participants, low VO_2max_ was associated with reduced levels of miR-151 and increased levels of miR-29a and miR-125a compared to male participants with high VO_2max_ (Figure 1B). In women, miR-210, let-7d and miR-21 were increased in subjects with low VO_2max_ compared to subjects with high VO_2max_ (ddCp>+/−0.50 and p<0.05) (Figure 1C).

**Table 1:** Baseline characteristics of the study populations.

	microRNA screening		microRNA validation	
Characteristics	Low VO_2max_ (n = 12)	High VO_2max_ (n = 12)	Low VO_2max_ (n = 38)	High VO_2max_ (n = 38)
Age	42.7 (1.6)	42.6 (1.7)	45.6 (3.2)	45.4 (3.3)
Gender (n = female/male)	6/6	6/6	19/19	19/19
VO_2max_ (ml/kg^0.75^/min)	104.2 (16.6)	151.2 (18.5)	101.1 (18.0)	145.2 (20.7)
Physical activity, Kurtze score [22]	3.5 (1.3)	3.5 (1.3)	3.8 (1.3)	3.8 (1.3)
Weight (kg)	72.6 (8.7)	80.2 (12.9)	78.1 (15.0)	79.5 (10.7)
Waist (cm)	87.8 (6.7)	89.0 (7.1)	90.4 (10.6)	89.6 (8.2)
Height (cm)	169.6 (6.2)	175.7 (10.6)	172.5 (10.1)	172.9 (7.7)
Hip (cm)	100.8 (4.9)	102.4 (6.6)	102.0 (7.1)	102.5 (5.8)
Body mass index (kg/m^2^)	25.2 (1.7)	25.8 (2.0)	26.1 (3.4)	26.6 (3.0)
Daily smoking (n)	1	1	4	2
Diabetes (n)	0	0	1	0
Systolic BP (mmHg)	126.8 (12.0)	123.3 (11.0)	125.5 (15.5)	128.1 (15.5)
Diastolic BP (mmHg)	69.3 (10.2)	72.9 (9.4)	75.9 (11.4)	74.3 (9.6)
Non-fasting glucose (mmol/l)	4.9 (1.1)	4.9 (0.8)	5.4 (1.3)	5.3 (1.1)
Non-fasting HDL-cholesterol (mmol/l)	1.3 (0.3)	1.3 (0.3)	1.3 (0.4)	1.4 (0.3)
Total cholesterol (mmol/l)	5.3 (1.0)	5.1 (0.8)	5.4 (1.3)	5.4 (0.9)
Serum triglycerides (mmol/l)	1.5 (0.5)	1.4 (0.7)	1.9 (1.3)	1.7 (1.0)
Serum CRP (mg/l)	0.7 (0.5)	0.9 (0.8)	2.3 (3.4)	0.9 (0.9)
Serum ALAT (u/l)	n<3^*^	n<3^*^	16.8 (5.3)	23.4 (12.7)
Serum ASAT (u/l)	n<3^*^	n<3^*^	22.5 (6.0)	21.6 (6.3)
FINDRISC Score	4.7 (3.1)	4.8 (2.3)	7.2 (3.5)	7.0 (3.6)

Values are presented as mean with standard deviation in bracles. Some of the values describe only the number of people having certain characteristics (gender, daily smokers and diabetics). VO_2max_: Maximal oxygen uptake, BP: Blood pressure, HDL: High-density lipoprotein, CRP: C - reactive protein, ALAT: Alanine aminotransferase, ASAT: Aspartate aminotransferase.

n<3^*^: Less than three subjects that had performed this test.

Based on the screening approach, the 7 significant miRs (miR-210, miR-21, miR-125a, miR-652, miR-151, miR-29a, and let-7d) were selected for further testing in the validation cohort. We also included miR-222 based on the significant correlation with self-reported habitual exercise intensity (r = 0.5, p<0.05). Additionally, three miRs that were stable expressed in the screening cohort (miR-16, miR-103, miR-425) were also included in the validation cohort for the purpose of endogenous normalization.

**Figure 2:**
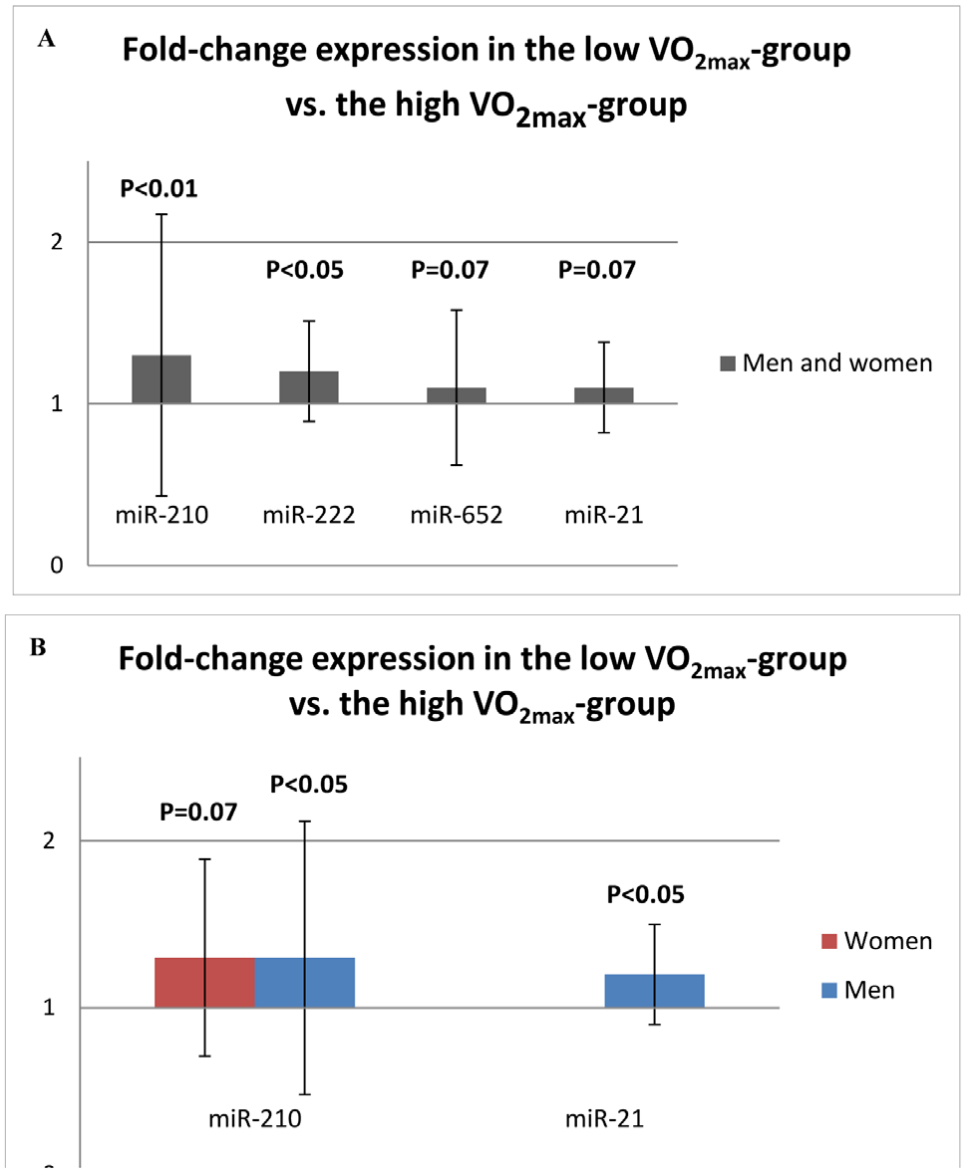
MicroRNAs with significant (or borderline significant) up-regulation in serum samples from subjects with low aerobic fitness compared to subjects with high aerobic fitness. A: In both men and women (n = 76), B: Both gender analyzed separately (n = 38 men, n = 38 women). VO_2max_: Maximal oxygen uptake. Error bars represent 1 standard deviation.

In the validation cohort, the level of circulating miR-210 and miR-222 were 30% and 20% higher, respectively in participants with low VO_2max_ compared to participants with high VO_2max_ (p<0.05) (Figure 2A). In addition, the miR-21 levels were 20% higher in male participants with low VO_2max_ compared to male participants with high VO_2max_ (p<0.05) (Figure 2B). The miR-125a levels were below the detection limit in most of the samples in the validation cohort and the group differences could therefore not be evaluated.

We observed a significant positive correlation between miR-21 and miR-210 (r = 0.42, p<0.0005), between miR-222 and miR-21 (r = 0.74, p<0.0005), and between miR-210 and miR-222 (r = 0.35, p<0.005) in the validation cohort.

### Correlations between miRs and Fitness Related Variables

A significant inverse correlation was found between VO_2max_ and miR-210 (n = 100, r = −0.35, p<0.001). Despite the observed group differences in miR-222 and miR-21, only a weak correlation was found between miR-21 and VO_2max_ (n = 100, r = −0.20, p<0.05) and no correlation was found between miR-222 and VO_2max_. No correlations were found between miR-210, miR-21 and miR-222 and exercise habits like frequency, intensity, and duration of self-reported regular physical activity, nor with self-reported time since last bout of exercise in the validation cohort.

Linear regression analysis showed that the serum level of miR-210 explained 12% of the variation in VO_2max_ (r^2^ = 0.12). When adding the serum levels of miR-21 and miR-222 to the analysis, these three miRs in combination explained 15% of the variation in VO_2max_. However, the best prediction model for VO_2max_ was obtained by using miR-210 alone. miR-210 levels in the lowest quartile increased the odds ratio by 10.4 times for having a high VO_2max_ (>120 ml/kg^0.75^/min).

### Correlations between miRs and Variables Related to CVD Risk

We observed significant correlations between the FINDRISC Score and miR-21 (n = 100, r = 0.37, p<0.001) and miR-222 (n = 100, r = 0.34, p<0.001). Assessing correlations with other CVD risk factors, we found a positive correlation between miR-21 and CRP (n = 51, r = 0.35, p<0.05) and between miR-210 and serum ASAT (n = 16, r = 0.78, p<0.0005). let-7d (n = 34 r = 0.48, p<0.005), and miR-103 (n = 62 r = 0.31, p<0.05), were also positively correlated with age in the validation cohort. In contrast, we found no correlations between the most common risk factors for CVD (BP, cholesterol, smoking habit, or obesity) and miR-21, miR-210 and miR-222 levels. Moreover, no correlations were found between the Framingham Risk Score or the NORRISK Score and miR-21, miR-210 and miR-222 levels.

### Targeted Pathways by Fitness-miRs

Bioinformatics may provide insight into signaling pathways that are targeted by different miRs [27]. Using bioinformatics, miR-21, miR-210 and miR-222 were predicted to target 611 genes. The KEGG pathway database recognized 138 of those genes. The signaling pathways that, based on prediction, were found to significantly segregate subjects with high and low VO_2max_ were the MAPK pathway, TGF-β pathway, B-cell receptor pathway, Wnt pathway, T-cell receptor pathway, mTOR pathway, p53 pathway, ErbB pathway, and VEGF pathway (p<0.05).

## Discussion

Despite the strong association between aerobic fitness level and the risk of future cardiovascular mortality, only limited information is currently available on serum biomarkers associated with aerobic fitness level. The present study demonstrates a potential value of circulating miRs as fitness biomarkers in a large cohort of healthy individuals. Our results especially identify miR-210 as a promising biomarker of aerobic fitness level based on increment in the low VO_2max_ group of both the screening and validation cohort and the significant correlation with VO_2max_.

Several previous studies have shown that induction of miR-210 is a hallmark of insufficient oxygen supply to tissues (hypoxia) [28]–[32]. The role of miR-210 during hypoxia is suggested to involve repression of mitochondrial metabolism, which is considered an important factor for aerobic fitness [33]. Tissue hypoxia plays a central role in the pathogenesis of ischemic disorders, such as myocardial infarction, stroke and peripheral artery disease [34]. A hypoxia-induced expression of miR-210 has been reported in several different organs, e.g. endothelium and the heart [29]–[31]. Furthermore, increased serum levels of miR-210 have been reported in patients with atherosclerosis and kidney disease [35], [36]. Since our cohort consists of predominantly healthy subjects, excluding subjects suffering from the most common diseases, the high level of miR-210 in subjects with low aerobic fitness may be an indication of hypoxic conditions, decreased mitochondrial metabolism and potentially subclinical disease in the cardiovascular system. Furthermore, since the high and low VO_2max_ group was similar regarding risk factors for CVD, miR-210 may represent an early marker of CVD risk even beyond traditional risk factors. The theory that miR-210 may provide information beyond traditional risk factors is strengthened by the lack of association with conventional cardiovascular risk scores.

In addition to miR-210, we also found significantly higher circulating levels of miR-21 and miR-222 in subjects with low aerobic fitness. miR-21 is one of the most highly and consistently up-regulated miRs during pathological cardiac hypertrophy and has received considerable attention as a marker of oxidative stress, inflammation, and pathology in both cardiomyocytes and endothelial cells [37]–[43]. In the present study, a significant positive correlation was found between serum levels of miR-21 and CRP, supporting the previous associations between miR-21 and inflammation. Since miR-21 is a highly ubiquitous miRNA found to be regulated by several conditions, we believe that this marker has to be included as a panel of markers to be usuful in prediction of fitness level and cardiovascular risk.

Less is known about circulating levels of miR-222, but strong expressions has been reported in vascular smooth muscles after vascular injury and in endothelial cells stimulated with angiogenetic factors [44], [45]. Furthermore, Baggish *et al* reported that vascular endothelial cells are capable of releasing high levels of both miR-21 and miR-222 in to the circulation [16].

The exact cellular sources of the measured miR-210, miR-21 and miR-222 in this study remains unclear and needs to be further elucidated in animal models and human subjects. However, a common denominator for these 3 miRs is the high expression in endothelial cells (Table 2) [29], [30], [35], [42], [44]–[46]. Theoretially, release of miR-210, miR-21 and miR-222 from the endothelium in healthy subjects with low VO_2max_ could be related to subclinical artherosclerosis, hypoxia or inflammation, hence providing a rationale for these miRs as novel early markers of CVD. Furthermore, additional organs could contribute to the circulating miR-210, miR-21 and miR-222 levels, including the heart. Although the participants in this study had no clinical overt heart disease, miR-210 and miR-21 have previously been found upregulated in cardiac hypertrophy, which can also be found in asymptomatic subjects [37], [47].

**Table 2:** Tissue of expression, associated cardiovascular diseases and biological processes linked to the differentially expressed microRNAs in this study.

miRs	Tissue expression	Cardiovascular diseases	Biological processes
**miR-210**	Endothelium [29], [30], [35], [46]	Atherosclerosis [35], [49]	Angiogenesis [29]
	Artherosclerotic plaque [49]	Myocardial infarction [50]	Hypoxia [28]–[33], [46]
	Heart [46], [50]		Proliferation [30]
			Mitochondrial metabolism [33]
**miR-222**	Endothelium [44], [45]	Atherosclerosis [35]	Angiogenesis [44]
			Proliferation [45]
**miR-21**	Endothelium [42]	Atherosclerosis [35], [49]	Cardiac hypertrophy [38], [41]
	Artherosclerotic plaque [49]	Heart failure [41]	Stress [42]
	Heart [40], [41]		Inflammation [43]
			Apoptosis [42]

miR: microRNA, VO_2max_: Maximal oxygen uptake.

Interestingly, a strong positive correlation was found between circulating levels of miR-210 and serum levels of ASAT. ASAT is commonly measured clinically as a marker of hepatocellular damage, but can also be released from injured myocardium. ASAT values are known to be influenced by exercise training and may remain significantly increased for several days after a bout of heavy exercise [48]. In our subjects the ASAT values were within the normal range. However the close correlation to miR-210 may indicate that the ASAT and miR-210 originates from the same organ. Since the correlation between ASAT and miR-210 is based on only 16 subjects, more research is needed to elucidate the association.

### Correlations between miRs and Variables Related to Fitness and CVD Risk

In this study we have matched the contrasting groups on physical activity level (based on the Kurtze score [22]), to avoid finding typical exercise induced miRs. Previous studies have shown that circulating levels of miR-21 and miR-222 are regulated by acute and long-term exercise training [16]. However, circulating levels of miR-210 have previously been reported to be unaffected both by acute and long-term exercise training [16]. In our study, no correlations were found between miR-210, miR-21 and miR-222 and exercise habits like frequency, intensity, or duration of self-reported regular exercise training. Additionally, no correlations were found between the three miRs and the time since the last exercise bout. Based on this, we can probably rule out the possibility that acute effects of exercise are responsible for our findings.

Surprisingly, no correlations were found between miR-21, miR-210 and miR-222 levels and important CVD risk factors such as BP, cholesterol, smoking habit, and obesity. However, miR-21 and miR-222 correlated with the FINDRISC score, which predicts the risk of being diagnosed with type 2-diabetes in the next ten years. As mentioned above, this finding strengthens our hypothesis that fitness biomarkers also have potential as markers of future cardiovascular health independent of traditional biomarkers.

### Target Genes and Pathways

miR-210 is considered the hypoxia-miR [28]. Thus, the high circulating levels of miR-210 in subjects with low VO_2max_, may suggest that hypoxia-induced pathways are activated. In line with this, the mirPath prediction tool identified the VEGF pathway, which is another important hypoxia pathway, to be differentially activated in the high and low VO_2max_ group. Interestingly, in endothelial cells miR-210 and the VEGF pathway seem to interact in the formation of new capillaries [30]. However, whether subjects with low aerobic fitness have increased activity in hypoxia- and angiogenesis pathways to compensate for the low oxygen uptake, will need confirmation in additional clinical and experimental studies.

**Figure 3:**
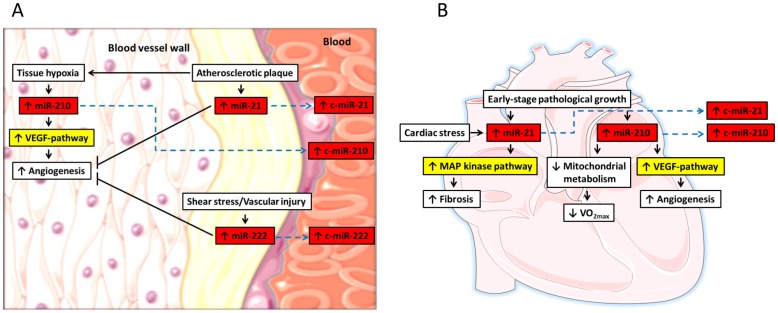
Potentially increased microRNAs and associated pathways in subjects with low maximal oxygen uptake (VO2max). The connections between the biological processes, microRNAs and the regulated pathways in A) the vessel wall and (B) the heart are based on the current knowledge in the literature. Red boxes illustrate the microRNAs that were increased in patients with low VO2max and the yellow boxes depict the associated pathways as identified by miR-Path analyses. Upward arrows inside the boxes illustrate up-regulation and downward arrows illustrate down-regulation. Blue dashed lines indicate the putative release of the microRNAs to the circulation. Pointed arrows illustrate activation and flat arrows illustrate inhibition. c-miR: Circulating microRNA, VEGF: Vascular endothelial growth factor, VO2max: Maximal oxygen uptake.

The pathway analysis also predicted that miR-21, miR-210 and miR-222 inhibit the expression of several key proteins in more general pathways associated with growth and development (MAPK, TGF-*β*, mTOR, ErbB and the Wnt pathways), the immune system (T and B cell signaling) and stress (p53 pathway). Potentially induced pathways in the vessel wall and the heart of subjects with low VO_2max_ are illustrated in Figure 3. The implications of this for the pathophysiology of subjects with low VO_2max_ is not clear and will need more detailed studies of specific pathways. We also acknowledge that currently only limited information is available regarding the origin of circulating miRs. Hence, extrapolating from miRs in the peripheral blood to functional aspects in specific organs should be done with caution. Still, the association to hypoxia- and angiogenesis-related pathways seems interesting and should be further explored.

To our knowledge only one previous study has described that circulating miRs may be associated with fitness measured as VO_2max_ [16]. This paper has reported a positive correlation between the circulating levels of miR-146a and VO_2max_ [16]. No such correlation was found in this study. However, this discrepancy may be explained by the differences in study-population as Baggish *et al* studied 10 male athletic subjects [16].

### Limitations

One limitation of this study is that the analyzed blood samples were collected from non-fasting individuals. However, the time since last meal was similar in both the high and low VO_2max_ group (3.1 hours). We therefore believe that food intake did not influence the miR results. Another limitation of the study is that 8 of the 100 participants reported to have performed exercise training the same day as blood sampling, spanning from 1 hour to 11 hours before the samples were collected. Baggish *et al* have previously shown that exercise training <24 hours before blood sampling can influence the levels of circulating miRs such as miR-222 and miR-21 independent of VO_2max_. However, removing these 8 participants from the statistical tests had no influence on the results. Furthermore, correlation analysis between miR-levels (miR-21, miR-210, miR-222) and self-reported time since last bout of exercise showed no correlations.

### Conclusion

The results from this study suggest a potential value of measuring circulating miRNAs as biomarkers of aerobic fitness. The results indicated that miR-210 was the best marker of VO_2max_-level, based on increment in the low VO_2max_ group in both the screening and validation cohort, and the significant correlation with VO_2max_-level. The lack of association between miR-210, miR-21, miR-222, and other fitness related variables indicates that these three miRs may have potential as new independent biomarkers of fitness level. Furthermore, the lack of association between miR-210, miR-21, miR-222 and traditional CVD risk factors suggest a potential of these miRs as early biomarkers of future cardiovascular health, and should be further studied. Potential bias involve that blood samples were collected from non-fasting individuals, and that 8 of the participants reported to have performed exercise training within 24 hours before sampling.
